# Universal Health Coverage on $15 per Capita: Somalia's Health Financing Crisis and the Limits of Humanitarian Dependency

**DOI:** 10.1002/puh2.70392

**Published:** 2026-09-30

**Authors:** Ahmed Abdinasir Abdulle, Ilyas Abdullahi Khalif, Aniso Mohamed Abdi, Yusuf Abdullahi Hubow, Nour Ahmed Dahir, Abdirahman Mohamed Adan, Sharmake Gaiye Bashir, Nuradin Abdullahi Sheikh Rashid

**Affiliations:** ^1^ Faculty of Health Sciences Mogadishu University Mogadishu Somalia; ^2^ Center for Health Research and Innovation Somali National University Mogadishu Somalia

**Keywords:** fragile and conflict‐affected states, health financing, humanitarian dependency, Somalia, universal health coverage

## Abstract

Universal health coverage (UHC) is unattainable without sustainable and equitable health financing. Somalia, operating with an estimated health expenditure of roughly $15 per capita, represents one of the starkest examples of the gap between global UHC ambitions and the realities of fragile‐state financing. This commentary examines how chronic underinvestment, heavy humanitarian dependency, fragmented donor financing and high out‐of‐pocket spending have undermined equitable healthcare access and financial protection in Somalia. Despite the lifesaving role of humanitarian assistance, prolonged reliance on externally driven and project‐based funding has weakened national stewardship, reinforced system fragmentation and limited long‐term resilience. We argue that Somalia's current financing architecture is fundamentally incompatible with sustainable UHC, particularly for rural, displaced and conflict‐affected populations. The article calls for urgent reforms centred on stronger domestic financing, pooled funding mechanisms, primary healthcare investment and alignment of external support with nationally led priorities. Somalia's experience highlights a broader global challenge: Without context‐sensitive financing reforms and sustained investment in fragile settings, UHC risks remaining an aspirational goal rather than a practical reality.

## 1 Introduction

Universal health coverage (UHC) is a core target of the Sustainable Development Goals (SDGs target 3.8), requiring that all people have access to the quality health services they need without financial hardship [1]. Yet fragile and conflict affected states are consistently off‐track: Somalia records one of the lowest UHC index scores globally (33.5/100), reflecting profound deficits in service coverage and financial protection [2]. This failure is rooted less in aspiration than in arithmetic. With per capita spending around the order of $15, Somalia's health system operates far below the levels observed even in low‐income settings, where average per capita spending is several times higher [2].

Decades of conflict, political fragmentation, recurrent droughts and floods and protracted humanitarian crises have entrenched a fragmented, donor‐driven health system, in which external aid and out‐of‐pocket (OOP) payments dominate and government contributes only a marginal share of total health expenditure [3]. Evidence of high OOP reliance, catastrophic spending and widespread unmet need underscores how such a financing model systematically excludes the poorest and undermines equity and access [3]. In this context, ‘$15 per person’ is not merely a budget line; it signifies an inability to fund even a minimal primary care platform, retain health workers or extend coverage to rural and displaced populations [4].

This commentary argues that current financing levels and structures are fundamentally incompatible with sustainable UHC in Somalia and similar fragile settings. It will examine Somalia's health financing crisis, interrogate the limits of humanitarian dependent systems and outline urgent reforms to build a more equitable, domestically anchored financing architecture capable of supporting universal, affordable care.

## 2 Somalia's Health Financing Crisis

Somalia's health financing architecture is structurally misaligned with the goals of UHC, combining extremely low public spending, heavy donor dependence and high OOP payments into a model that systematically reproduces exclusion. Government spending accounts for only about 5% of total health expenditure, with the vast majority of resources provided by external partners and households, leaving the state with minimal leverage over service provision, equity or quality [5]. Per capita health spending is notably low by international standards, and Somalia's overall UHC index score of 33.5 is the lowest among 120 countries assessed, underscoring the depth of underinvestment relative to regional and global benchmarks [2].

This fiscal fragility is rooted in decades of conflict and institutional erosion, which have constrained revenue generation, hollowed out regulatory capacity and contributed to the dominance of the private health sector, which now provides about 80% of curative care services in Somalia [6, 7]. Donor funding, while lifesaving, flows largely off‐budget through vertical projects and parallel supply and information systems, fragmenting risk pools and creating territorial islands of coverage around humanitarian priorities rather than population need [3]. OOP payments account for approximately 44.9% of total health expenditure—well above global and sub‐Saharan African averages—whereas health insurance coverage remains extremely limited, with a recent Somalia‐specific study reporting coverage of only 3.5% [8]. WHO likewise reports that Somalia has no national health insurance scheme and that only 2% of households in the 2020 Somali Health and Demographic Survey used private health insurance [7]. These limited financial protection mechanisms contribute to catastrophic health spending and foregone care, particularly among poorer households [8].

Chronic underfunding and fragmented purchasing translate directly into skeletal primary care networks, shortages of essential medicines and severe workforce deficits, especially in rural and displacement affected areas [2, 3]. These weaknesses undermine routine maternal, newborn and child health services; depress vaccination coverage and erode system resilience to epidemics and climate‐related shocks, entrenching a cycle in which financial fragility and health inequities are mutually reinforcing rather than progressively resolved [2, 5].

## 3 Why UHC Remains Out of Reach

Somalia's current financing model structurally precludes meaningful progress toward UHC. Public spending accounts for less than 5% of health expenditure, with the system dominated by external aid and OOP payments, in a context of one of the lowest per capita health outlays globally [3, 6]. This underinvestment produces a UHC service and financial protection index of just 33.5—the lowest among 120 countries—signalling systemic failure to deliver even basic services without hardship [2]. Extreme reliance on household payments (around 45% of total health spending) translates into catastrophic expenditure for a quarter of Somali households and widespread forgone care [8].

At such low spending levels, primary healthcare remains thinly resourced and urban biased, with severe workforce shortages and private sector dominance leaving rural communities and internally displaced persons particularly underserved [2, 6]. Preventive and promotive services, including immunisation and sexual and reproductive health, are chronically underfunded, sustaining high burdens of avoidable maternal, newborn and child morbidity and mortality [3]. Weak, fragmented financing also undermines infectious disease control, non‐communicable disease management and emergency preparedness, despite global calls to integrate UHC and health security in fragile settings [9].

Global UHC frameworks frequently presuppose stable governance, robust fiscal space and institutional capacity to pool funds and purchase services strategically assumptions that are misaligned with Somalia's conflict affected, donor‐dependent reality [10]. The result is an inequitable financing architecture that systematically privileges wealthier and better educated groups, deepens rural urban and gendered disparities and keeps Somalia off‐track for SDG 3, with implications for equity across fragile states more broadly [2].

## 4 The Cost of Humanitarian Dependency

Somalia's health system has been rebuilt largely through humanitarian and donor funding, with government contributing only a small fraction of total health spending, leaving UHC indicators among the lowest globally [4, 6]. Although this aid has been indispensable in expanding immunization, maternal child health services and basic primary care in a context of protracted crisis, its design and financing modalities embed structural weaknesses that undermine long‐term system resilience and progress toward UHC [2, 6].

Short donor cycles and projectized funding have entrenched parallel systems off‐budget flows, separate supply chains and stand‐alone information platforms limiting the Ministry of Health's capacity to steer priorities, pool risks and plan beyond emergency horizons [3, 11]. Evidence from fragile and conflict‐affected settings (FCAS) shows that such fragmentation reduces efficiency, weakens equity and obstructs strategic purchasing, particularly for primary healthcare (PHC), the core platform for UHC [6]. In Somalia, this translates into service gaps, geographic inequities and heavy reliance on OOP spending, with high risks of catastrophic expenditures among the poorest [2, 4].

Humanitarian‐centred models, focused on life‐saving packages and vertical programmes, tend to stabilize crisis mortality without systematically investing in integrated PHC, workforce retention or domestic fiscal space, thereby perpetuating an ‘emergency default’ in service delivery [4, 11]. Comparative and regional analyses suggest that when external resources bypass national systems and payrolls, they can destabilize health worker labour markets, dilute accountability and blunt incentives for domestic resource mobilization [11, 12].

For Somalia, transitioning from humanitarian dependency to sustainable UHC requires deliberate reorientation of aid: increasing on‐budget, pooled and PHC first investments; aligning external funds with national strategies; and using humanitarian finance explicitly as a bridge toward nationally led, equity‐oriented health financing reforms [3, 11].

## 5 Financing Reform and Policy Solutions

Financing reform for UHC in Somalia must start from the recognition that a system reliant on OOP payments, minimal public funding and fragmented aid is structurally incompatible with equitable UHC. With government spending estimated at around 5% of total health expenditure and OOP accounting for nearly 45%, a quarter of Somali households already face catastrophic health spending, undermining both access and financial protection [2, 3]. Any viable reform strategy therefore needs to rebalance the system toward pooled, predictable and progressively raised public resources.

Somalia's substantial diaspora remittance flows also warrant consideration within the broader health‐financing landscape. Remittances, estimated at about 20.6% of GDP in 2022 and supporting a large share of Somali households, could potentially complement formal financing through carefully regulated remittance‐linked prepayment or insurance mechanisms; however, such private transfers should supplement rather than substitute for sustainable public financing and equitable risk pooling [13, 14].

Incrementally increasing domestic public health expenditure largely through strengthened, progressive tax‐based financing and careful use of ‘innovative’ earmarked taxes can expand fiscal space, but evidence from other African settings cautions that such mechanisms raise modest revenue and require strong governance and administrative capacity to avoid distortion and leakage [5, 9]. In Somalia, this implies sequencing reforms with broader public financial management and anti‐corruption efforts, alongside transparent expenditure tracking across health system elements to build trust and accountability [3, 15]. Consolidating highly fragmented donor funding into pooled arrangements aligned with a national UHC strategy, as called for in FCAS, is essential to reduce duplication, enable strategic purchasing and shift incentives toward PHC and community health systems rather than ad hoc humanitarian projects [2, 5].

Reorienting financing towards PHC through protected budget lines, needs‐based resource allocation and population‐based provider payment can address Somalia's severe inequities and under‐provision of essential services, particularly in rural and conflict affected areas [5, 15]. Long‐term international partnerships remain indispensable but should explicitly support resilience: co‐financing recurrent PHC and community health worker platforms, subsidizing prepayment schemes for the poor and investing in digital health, data systems and workforce capacity rather than episodic emergency campaigns [2, 5]. Experience from other low and lower middle‐income countries shows that compulsory prepayment, merged risk pools and strategic purchasing are feasible even in constrained environments when anchored in clear political commitments and gradual implementation pathways [10, 16]. For Somalia, embedding financing reform within wider state‐building strengthening institutions, regulatory capacity and inclusive governance is not a luxury but a precondition for sustainable UHC, and a critical test case for the global pledge to achieve equitable UHC in fragile states [2, 17].

## 6 Global Implications for UHC in Fragile States

Somalia's health financing emergency epitomizes a structural fault line in the global UHC agenda rather than an outlier. With the lowest UHC index score among 120 countries and severe financial hardship deterring care‐seeking, Somalia illustrates how conflict, poverty and institutional fragility systematically depress service coverage and financial protection despite global commitments to ‘leave no one behind’ [2]. Similar patterns of inequitable access and catastrophic expenditures in other FCAS show that Somalia's predicament is emblematic of broader governance and financing crisis, not simply a country specific failure [5].

Current UHC and SDG frameworks largely presume functioning institutions, predictable fiscal space and integrated health systems, conditions that are routinely absent in FCAS [2]. Evidence from conflict‐affected African settings and subnational analyses in fragile states demonstrates that proximity to violence magnifies wealth‐ and education‐related disparities in primary care access, reinforcing exclusion from SDG 3 progress [18]. At the same time, humanitarian financing remains volatile, increasingly underfunded and heavily channelled through vertical, off‐budget programmes, entrenching parallel systems and weakening state stewardship, as seen in Somalia's prolonged aid driven model [11].

Realigning global health financing with the realities of fragility requires moving beyond short‐term, emergency‐oriented aid toward long‐term, equity‐focused and resilience‐centred investments in PHC, domestic capacity and governance [5, 11]. Pooled funding, stronger country ownership and risk pooling mechanisms that explicitly incorporate conflict and displacement are critical to prevent FCAS from remaining perpetual laggards in UHC and SDG trajectories [2, 5].

## 7 Conclusion

Somalia's health financing emergency exposes the stark incompatibility between rhetorical commitments to UHC and the reality of attempting to fund an entire health system on roughly $15 per person. This commentary has argued that such chronic underinvestment is not only a profound national crisis, but also a critical stress test for global UHC aspirations in fragile and conflict‐affected settings. A model that depends primarily on short‐term humanitarian aid and fragmented external projects cannot deliver sustained access to essential services, protect households from financial hardship or build resilient systems capable of withstanding recurrent shocks. Transformative financing reforms anchored in increased and more predictable domestic resources, pooled and equity‐oriented international support and strengthened governance are therefore urgent, not optional. Looking ahead, achieving UHC in Somalia and comparable contexts will require sustained political commitment, fairer global financing rules and context‐sensitive policy design that explicitly centres fragility rather than treating it as an afterthought. Without such recalibration, UHC will remain an aspirational slogan rather than a realizable guarantee of health for all.

## Author Contributions

**Ilyas Abdullahi Khalif** conceptualized this topic. **Nuradin Abdullahi Sheikh Rashid** and **Ahmed Abdinasir Abdulle** supervised this project. **Yusuf Abdullahi Hubow** and **Nour Ahmed Dahir** conducted literature review. **Aniso Mohamed Abdi** and **Abdirahman Mohamed Adan** wrote the first draft. **Sharmake Gaiye Bashir** revised and edited this manuscript. All authors approved the final version of the manuscript.

## Funding

The authors have nothing to report.

## Conflicts of Interest

The authors declare no conflicts of interest.

## Data Availability

Data sharing is not applicable to this article as no datasets were generated or analysed during the current study.

## References

[1] WHO , Tracking Universal Health Coverage 2023 Global Monitoring Executive Summary (World Health Organization, 2023).

[2] Z. Nikoloski , M. M. Mohamoud , and E. Mossialos , “Universal Health Coverage in Fragile and Conflict‐Affected States: Insights From Somalia,” International Journal for Equity in Health 24 (2025): 125, 10.1186/s12939-025-02486-3. PMC12060528 40336100

[3] A. Y. Ahmed , F. A. Nor , M. Y. Ahmed , et al., “Universal Health Coverage in Somalia: Charting the Path to Equitable Healthcare Financing and Governance,” Health 15 (2023): 1298–1317, 10.4236/health.2023.1511085.

[4] K. Hanson , N. Brikci , D. Erlangga , et al., “The Lancet Global Health Commission on Financing Primary Health Care: Putting People at the Centre,” Lancet Global Health 10 (2022): e715–e772, 10.1016/S2214-109X(22)00005-5. PMC9005653 35390342

[5] B. Bogale , S. Scambler , and J. E. Gallagher , “Health System Strengthening in Fragile and Conflict‐Affected States: A Review of Systematic Reviews,” PLoS ONE 19 (2024): e0305234, 10.1371/journal.pone.0305234. PMC11178226 38875266

[6] A. S. Said and D. I. Kicha , “Implementing Health System and the New Federalism in Somalia: Challenges and Opportunities,” Front Public Health 12 (2024): 1205327, 10.3389/FPUBH.2024.1205327. PMC10867962 38362207

[7] World Health Organization Regional Office for the Eastern Mediterranean , Understanding the Private Health Sector in Somalia (WHO, 2024).

[8] K. M. Mohamud , S. Y. Warei , and A. H. A. Abubakr , “Burden or Benefit? Unveiling the Impact of Out‐of‐Pocket Health Expenditures in Somalia's Healthcare System,” Health Economics Review 16 (2025): 5, 10.1186/S13561-025-00703-X. PMC12821054 41385027

[9] H. C. Karamagi , D. Njuguna , S. N. Kidane , et al., “Financing Health System Elements in Africa: A Scoping Review,” PLoS ONE 18 (2023): e0291371, 10.1371/journal.pone.0291371. PMC10499258 37703243

[10] S. C. Ifeagwu , J. C. Yang , R. Parkes‐Ratanshi , and C. Brayne , “Health Financing for Universal Health Coverage in Sub‐Saharan Africa: A Systematic Review,” Global Health Research and Policy 6 (2021): 8, 10.1186/s41256-021-00190-7. PMC7916997 33641673

[11] S. A. Hussein , A. J. A. Ali , M. M. Osman , et al., “Prolonged Multisectoral Aid‐Driven Reliance on Health Systems and Governance Post‐Conflict Era in Somalia,” Tropical Medical and Health 54 (2026): 10, 10.1186/S41182-025-00861-Y. PMC12781686 41508251

[12] N. I. Dirie , M. M. Ahmed , Y. B. Abdullahi , et al., “Transforming Health in Post‐Conflict Somalia: Priorities From a Multi‐Stakeholder Roundtable on the 2025–2029 National Plan,” Journal of Healthcare Leadership 17 (2025): 459–468, 10.2147/JHL.S541966. PMC12439833 40964046

[13] Y. Chen and K. B. Ajide , “Remittances, Health Financing Systems, and SDG Three Outcomes in Africa,” Sustainable Development (2026): 1–22, 10.1002/SD.70874.

[14] World Bank Group , Country Private Sector Diagnostic: Creating Markets in Somalia—Unlocking Private Sector‐Led Growth at a Critical Juncture of Somalia's Development (World Bank Group, n.d.), https://documents.worldbank.org/en/publication/documents‐reports/documentdetail/099720301202532976.

[15] A. Asante , W. S. K. Wasike , and J. E. Ataguba , “Health Financing in Sub‐Saharan Africa: From Analytical Frameworks to Empirical Evaluation,” Applied Health Economics and Health Policy 18 (2020): 743–746, 10.1007/s40258-020-00618-0. PMC7609366 33145665

[16] A. H. Kaiser , N. Rotigliano , S. Flessa , B. Ekman , and J. Sundewall , “Extending Universal Health Coverage to Informal Workers: A Systematic Review of Health Financing Schemes in Low‐ and Middle‐Income Countries in Southeast Asia,” PLoS ONE 18 (2023): e0288269, 10.1371/journal.pone.0288269. PMC10335706 37432943

[17] M. Y. Lim , H. F. Kamaruzaman , O. Wu , and C. Geue , “Health Financing Challenges in Southeast Asian Countries for Universal Health Coverage: A Systematic Review,” Archives of Public Health 81 (2023): 148, 10.1186/s13690-023-01159-3. PMC10433621 37592326

[18] M. Ramadan , H. Tappis , M. V. Uribe , and W. Brieger , “Access to Primary Healthcare Services in Conflict‐Affected Fragile States: A Subnational Descriptive Analysis of Educational and Wealth Disparities in Cameroon, Democratic Republic of Congo, Mali, and Nigeria,” International Journal for Equity in Health 20 (2021): 253, 10.1186/S12939-021-01595-Z. PMC8665620 34895244

